# Perlecan Domain-V Enhances Neurogenic Brain Repair After Stroke in Mice

**DOI:** 10.1007/s12975-020-00800-5

**Published:** 2020-04-07

**Authors:** Amanda L. Trout, Michael P. Kahle, Jill M. Roberts, Aileen Marcelo, Leon de Hoog, Jeffery A. Boychuk, Stephen L. Grupke, Antonio Berretta, Emma K. Gowing, Carie R. Boychuk, Amanda A. Gorman, Danielle N. Edwards, Ibolya Rutkai, Ifechukwude J. Biose, Hatsue Ishibashi-Ueda, Masafumi Ihara, Bret N. Smith, Andrew N. Clarkson, Gregory J. Bix

**Affiliations:** 1 Sanders-Brown Center on Aging, University of Kentucky, Lexington, KY USA; 2 Department of Neurology, University of Kentucky, Lexington, KY USA; 3 Department of Neuroscience and Experimental Therapeutics, Texas A&M Health Science Center College of Medicine, Bryan, TX USA; 4 Department of Neuroscience, University of Kentucky, Lexington, KY USA; 5 Department of Physiology, University of Kentucky, Lexington, KY USA; 6 Department of Neurosurgery, University of Kentucky, Lexington, KY USA; 7 Department of Anatomy, Brain Health Research Center and Brain Research New Zealand, University of Otago, Dunedin, New Zealand; 8 Clinical Neuroscience Research Center, Department of Neurosurgery, Tulane University School of Medicine, New Orleans, LA USA; 9 Tulane Brain Institute, Tulane University, New Orleans, LA USA; 10 Department of Pathology, National Cerebral and Cardiovascular Center, Suita, Japan; 11 Department of Neurology, National Cerebral and Cardiovascular Center, Suita, Japan

**Keywords:** Perlecan, Integrin, Neurogenesis, Stroke, Neurorepair

## Abstract

The extracellular matrix fragment perlecan domain V is neuroprotective and functionally restorative following experimental stroke. As neurogenesis is an important component of chronic post-stroke repair, and previous studies have implicated perlecan in developmental neurogenesis, we hypothesized that domain V could have a broad therapeutic window by enhancing neurogenesis after stroke. We demonstrated that domain V is chronically increased in the brains of human stroke patients, suggesting that it is present during post-stroke neurogenic periods. Furthermore, perlecan deficient mice had significantly less neuroblast precursor cells after experimental stroke. Seven-day delayed domain V administration enhanced neurogenesis and restored peri-infarct excitatory synaptic drive to neocortical layer 2/3 pyramidal neurons after experimental stroke. Domain V’s effects were inhibited by blockade of α2β1 integrin, suggesting the importance of α2β1 integrin to neurogenesis and domain V neurogenic effects. Our results demonstrate that perlecan plays a previously unrecognized role in post-stroke neurogenesis and that delayed DV administration after experimental stroke enhances neurogenesis and improves recovery in an α2β1 integrin-mediated fashion. We conclude that domain V is a clinically relevant neuroprotective and neuroreparative novel stroke therapy with a broad therapeutic window.

**Electronic supplementary material:**

The online version of this article (10.1007/s12975-020-00800-5) contains supplementary material, which is available to authorized users.

## Introduction

Stroke is a leading cause of death and disability [1]. The only FDA-approved pharmaceutical for ischemic stroke, tissue plasminogen activator (tPA), has a narrow therapeutic window [2]. While thrombectomy is now standard of care, clinical outcome lag behind improved recanalization rates [3]. Many experimental stroke therapies also have a limited therapeutic window and/or have failed in clinical trials [4, 5], suggesting a critical need for stroke therapies with a broader therapeutic window. We recently demonstrated that administration of recombinant perlecan domain V (DV) 24 h after transient middle cerebral artery occlusion (MCAo) in mice and rats promotes the brain neurorepair. Following photothrombotic stroke, DV was also neuroprotective and improved functional outcome in both young and aged mice [6]. DV crosses the blood-brain barrier and drives VEGF-mediated processes of neuroprotection and angiogenesis [7], while peri-infarct astrogliosis is increased acutely but suppressed chronically [8]. Together, this suggests DV may be a novel therapeutic for stroke.

DV’s parent molecule perlecan is a > 400 kDa heparan sulfate proteoglycan consisting of five protein domains (I–V) and several glycosaminoglycan chains, and resides in the extracellular matrix (ECM) of basement membranes (BMs) [9]. Perlecan plays a critical role in maintaining BM integrity and vasculo- and angiogenesis [10], mediating epithelialization, supporting adhesive separation and maintenance of neuroepithelium [11], and is found in human brain neuroectoderm and capillary BMs as early as gestational week six [12]. Importantly, complete perlecan knockout is embryonic lethal, and mice have brain atrophy associated with reduced cell proliferation, hampered migration of developing neurons, and impaired neurogenesis [13, 14]. Conversely, perlecan treatment promotes in vitro neural stem/progenitor cell proliferation and neuritogenesis [15]. Collectively, this suggests a key role of perlecan in developmental neurogenesis.

Following stroke, subventricular zone (SVZ) neurogenesis increases and migration of neuroblasts is redirected towards the damaged area, where they provide trophic support and may replace lost neurons. Although few new neurons reach and survive in the damaged area naturally, therapies may be capable of boosting neurogenesis to a level that provides functional improvement [16–18]. Importantly, neurogenesis is causally linked with angiogenesis in neurovascular niches, and migration of these newly born neurons is facilitated by blood vessel scaffolds [19].

Following stroke, extensive proteolysis and remodeling occurs in the ECM and BM resulting in robust generation of DV cleaved from perlecan [7, 20]. Our use of recombinant DV as a post-stroke therapy is aimed at enhancing the brain’s own protection and repair responses. The importance of perlecan/DV is reinforced by our previous work demonstrating that perlecan deficient mice experience larger infarcts and less reparative angiogenesis than littermate wild-type (WT) controls after transient MCAo [7]. Likewise, Nakamura et al. independently demonstrated that a differently generated perlecan knockout mouse also experienced larger infarcts than WT controls after transient MCAo [21]. Given perlecan’s implication in developmental neurogenesis, the spatiotemporal generation of DV following stroke, and its driving of reparative angiogenesis, we hypothesize that DV plays an important role in post-stroke neurogenesis by promoting neurogenic repair, even when treatment is delayed for 7 days. Therefore, we investigated the potential of DV to have a broad therapeutic window in experimental ischemic stroke models.

## Materials and Methods

### Adherence to STAIR Criteria

DV efficacy was tested in multiple stroke models, two age groups, and in multiple labs as suggested by the STAIR recommendations for preclinical stroke research [22]. Likewise, all studies were randomized and the experimenter(s) blinded to treatment conditions to ensure unbiased data collection and data processing.

### Human Brain Tissue Immunohistochemistry

Postmortem brain tissues from autopsy-confirmed cases of ischemic stroke and controls were obtained from the National Cerebral and Cardiovascular Center (Suita, Japan). Informed consent was secured for all subjects. Experiments involving human subjects were performed in accordance with relevant guidelines and regulations and were approved by the ethics committee of National Cerebral and Cardiovascular Center. Slides containing 6-μm paraffin embedded–formalin fixed human brain sections were placed at 37 °C overnight, deparaffinized in xylene, and rehydrated to distilled water (diH_2_O). Antigen was unmasked in heated in citrate buffer (pH 6) and washed in diH_2_O. Endogenous peroxidase activity was blocked with 3% (*v*/*v*) H_2_O_2_ in methanol, slides were rinsed in diH_2_O and PBS, and then blocked in 10% (*v*/*v*) normal goat serum for 1 h. Primary antibody (all antibodies listed in Online Resource Table 1) was added overnight at 4 °C. Slides were washed in PBS, incubated with a secondary antibody for 1 h, and washed in PBS. Signal was amplified using avidin-biotin substrate for 60 min, developed with DAB chromogen, and rinsed in diH_2_O. Slides were counterstained with hematoxylin, dehydrated in sequential water to ethanol, cleared in xylene, and mounted. All slides were scanned on the AperioScanScope XT digital slide scanner at × 40 and stitched together to create a single × 1 micrograph of the tissue. Images were viewed on Imagescope and infarcted regions were positively identified by a neuropathologist. For each sample, minimally 6 peri-infarct images were collected and normalized in Photoshop to match background of control samples between multiple days of imaging. Images were further analyzed with ImageJ (NIH) to separate DAB colors by deconvolution to calculate optical density. All images were normalized to day-specific controls.

### Tandem Ipsilateral Common Carotid/Middle Cerebral Artery Occlusion Stroke Model

Mice were housed in a climate-controlled room on a 14/10 h light/dark cycle and food and water were provided ad libitum. WT (C57Bl/6 J) and pln^−/−^ [7, 23] male mice (3 months old) were subjected to transient tandem ipsilateral common carotid artery (CCA)/middle cerebral artery (MCA) occlusion (MCAo) for 60 min as previously described [7]. Briefly, a small burr hole was made in the skull to expose the MCA and a 0.005 in. metal wire was placed under the artery. Slight elevation of the metal wire causes visible occlusion of the MCA. The CCA was then isolated and occluded using an aneurysm clip. Study inclusion criteria of diminished (> 80%) and reperfused (75% of baseline) blood flow was confirmed with laser Doppler perfusion monitor (Perimed). Animals were excluded from the study if the CCA or MCA was punctured during the procedure; they died during or following surgery, or needed medical euthanization. Historically, we see about a 5 % intraoperative mortality with no postoperative mortality with our MCAo model, similarly to what is previously published [24]. Surgical sham controls were performed without the occlusion of the MCA or CCA.

Mice were randomly assigned to a no treatment control, vehicle (PBS), or DV-treated (2 mg/kg) group receiving IP injections every 3 days from post-stroke (or sham) day (PSD) 7 to 19. Human recombinant DV was purified as previously described [7]. PBS was used as a vehicle control as prior studies showed that heat-inactivated DV has no effect on animal vital signs, blood gases, electrolytes, infarct volume, or angiogenic neurorepair [7]. WT mice were sacrificed on PSD 21 while pln^−/−^ mice were sacrificed on PSD 14. Brains were extracted, flash frozen, and sectioned (20 μm).

### Photothrombosis Stroke Model

Male C57Bl/6 J mice, aged (24 months) and young (2–3 months), were subjected to focal ischemic stroke by photothrombosis as previously described [25, 26]. Briefly, Rose Bengal (200 μL of a 10 mg/mL solution) was administered IP 5 min before 15 min illumination through the intact skull over the motor cortex affecting sensory forelimb and hindlimb as well as primary forelimb and hindlimb cortical areas [27]. The photothrombotic model has previously reported very low mortality (reviewed in [28]).

Aged mice were randomly assigned to receive IP injections of DV (2 mg/kg) or vehicle (PBS) beginning 6 h after injury, then on PSD 1, 2, 4, and 6, and sacrificed on PSD 7 by paraformaldehyde perfusion. The tissue was post-fixed and cryoprotected in sucrose before sectioning (30 μm). Alternatively, young mice were randomly assigned to receive IP injections of DV (2 mg/kg) or vehicle (PBS) beginning 6 h after injury, then on PSD 1, 2, 4, 6, 8, 10, 12, and 14. Starting on PSD 3, the two groups of 16 mice were further divided into four groups of 8 receiving an α2-blocking antibody (1 ml of a 1 mg/ml solution) or an IgG control antibody (1 ml of a 1 mg/ml solution) directly into the brain at AP + 0.00, ML – 1.2, DV − 2.5. The Hamilton syringe was left in place for 5 min post-injection to allow for proper diffusion. Mice were sacrificed 6 week post-stroke and perfused with paraformaldehyde, and brain tissue was processed as described above.

### Behavioral Testing

Behavioral testing took place prior to (baseline) MCAo surgery and on PSD 1, 4, 6, 8, 11, 13, 15, 18, and 20. Mice were placed on the rotor-rod (San Diego Instruments) for 5 min with increasing acceleration (0–40 rpm, 3 trials) measuring total distance (cm). Grip strength (force, g) was evaluated with a digital force-gauging apparatus (San Diego Instruments). Each animal underwent 3 trials per testing day.

Mice that received a photothrombotic stroke were evaluated on the cylinder and grid-walk test. Testing took place prior to (baseline) surgery and then on weeks 1, 2, 4, and 6 following stroke. The mice were placed in a Plexiglas cylinder for 5 min and time spent using the left, right, or both paws were recorded. For the grid-walk, mice were allowed to walk over a metal grid, and the number of foot faults and total steps taken were measured and are reported (percent of foot faults relative to total steps taken) as previously described [27].

### Histology

H&E staining was performed with the Leica Autostainer XL using standard methods. H&E dysmorphic areas were defined as regions with loss, lower density, smaller, irregular shaped nuclei, or irregular tissue patterning from surrounding areas. Areas, 3 individual tracings per image, were calculated using the ImageJ (NIH) free-hand selection tool. Fixed regions of interest (ROI) were selected based on historic infarct areas for our MCAo model. Images analyzed in Photoshop (thresholded for pixel intensity).

### Immunofluorescence

Tissue sections were fixed with 50:50 cold acetone/methanol and blocked in 5% BSA for 1 h. Primary antibody was added overnight at 4 °C. Sections were washed, incubated with the respective secondary antibody for 1 h, washed, and coverslipped with fluorescent mounting media containing DAPI. Images were captured using a Nikon Eclipse Ti or Olympus BX51 microscope and software. Images were analyzed for antibody-specific positive staining using Photoshop (thresholded and recorded positive pixels). Results are from 2 to 3 sections per animal. Confocal images were taken on a NikonA1 and processed in FIJI. The integrated density value (IDV) was measured in all 4 ROIs across channels using the multi-measure tool in ROI manager and divided by the average DAPI cell number IDV.

Cells were fixed (4% paraformaldehyde), washed, blocked in 10% BSA, and incubated overnight at 4 °C with primary antibody. Cells were incubated with fluorescent secondary antibody for 30 min at 37 °C and counterstained with DAPI. Images were captured as above.

### Neocortical Slice Preparation and Electrophysiology

All methods, including contents of artificial cerebrospinal fluid (ACSF) and internal pipette solutions, have been reported previously unless otherwise stated [29, 30]. PSD21 coronal neocortical slices (350 μm), each dorsal half of the hemisphere located ipsilateral to injury or sham injury, were isolated into a holding chamber containing ACSF.

After equilibration (≥ 1 h), slices were recorded in a chamber on an upright, fixed-stage microscope equipped with infrared, differential interference contrast optics (IR-DIC; Olympus BX51WI), where ACSF (32–34 °C) was continually superfused. Whole-cell patch-clamp recordings were performed from neocortical layer 2/3 pyramidal cells, targeting the agranular cortex. Glass recording pipettes were filled with 130 mM K^+^-gluconate [29]. Neural activity was recorded using an Axon Multiclamp 700B patch-clamp amplifier (Molecular Devices), acquired at 10–20 kHz and low-pass filtered at 5 kHz using a Digidata 1440A digitizer and the pClamp software (v10.3; Molecular Devices). Open tip resistance was 2–5 MΩ, seal resistance was 1–5 GΩ; series resistance was uncompensated and was required be < 25 MΩ with < 20% change during the recording (mean = 15.07 ± 0.65 MΩ, *n* = 34). No correction was applied to a liquid junction potential of − 7 mV.

Cells were allowed to acclimate ≥ 5 min following establishment of whole-cell configuration. Intrinsic properties were measured first. Membrane potential was recorded in *I* = 0 mode and analyzed in a 15-s interval. Input resistance was measured as the slope of the linear component of steady-state voltage responses to a series of current steps (− 40 pA steps of 500 msec; range + 160 to − 200 pA) using pClamp. Action potential thresholds were tested with minimum depolarizing current steps (+ 50 pA steps of 500 msec) and analyzed using Minianalysis (6.0.3; Synaptosoft). In the bath, bicuculline methiodide (30 μm), type A GABA receptor antagonist, was applied to isolate sEPSCs and tetrodotoxin (1 μm) to isolate mEPSCs in voltage-clamp mode with a voltage command of − 65 mV. Synaptic currents were analyzed off-line with Minianalysis and detected at × 3 the root mean squared noise level for each recording. A single-exponential EPSC decay time constant was also measured using a fraction of peak to find a decay time setting of 0.37 with no weighted adjustments. All electrophysiological parameters were averaged across neurons (i.e., *n* = number of neurons).

### BrdU Incorporation

Mice were injected with BrdU (IP, 100 μL of 10 mg/kg) following MCAo on PSD 7–13 and again on PSD 20–21. Brain sections were fixed with 4% paraformaldehyde for 2 h, washed, and incubated in 1 N HCL for 10 min on ice. This was followed by 2 N HCl for 10 min at room temperature and then 20 min at 37 °C. After the acid washes, 0.1 M borate buffer was added for 12 min at room temperature. Brain slices were washed, blocked, and incubated with primary antibody against BrdU at 4 °C for 48 h. Fluorescent secondary antibody was added for 1 h, then, sections were coverslipped and imaged as above.

### Neurosphere Cell Culture

Neurosphere-dissociated cells (STEMCELL Technologies) were cultured in complete proliferation media (STEMCELL Technologies, NeuroCult NSC Basal Medium) with NeuroCult NSC Proliferation Supplement and recombinant human epidermal growth factor (rhEGF, 20 ng/mL) in T-75 cm^2^ flasks at 37 °C and 5% CO_2_.

### Neurosphere Differentiation

Differentiation assays were performed in 24-well plates at 2.5 × 10^5^ cells/well. Neurospheres were allowed to adhere for 30 min prior to α2 nAb treatment and then were allowed to incubate for 15 min prior to DV or vehicle treatment. Cells remained in culture in differentiating media conditions (stem cell) until immunocytochemical analysis on DAP 6.

### qPCR

Experiments were and preserved in Trizol Reagent at HAP 6 for migration experiments and on DAP 3 for differentiation experiments. RNA was extracted using the PureLink™ RNA Mini Kit and converted into cDNA using the High-Capacity cDNA Reverse Transcription Kit (Applied Biosystems) using manufacturers’ instructions. Real-time PCR was performed with TaqMan fast advanced master mix (Applied Biosystems), with normalization to the housekeeping gene 18 s, using the ViiA™ 7 qPCR system (Applied Biosystems). Fold changes were determined using the ΔCt method [31].

### Neurite Extension Assays

E16 C57Bl/6 J mice cortices were isolated as described previously [32]. The neurons were then plated in 96-well plates at 100,000 cells/well in DMEM/B27 media and incubated overnight at 37 °C. Treatment conditions were then applied to the cells without B27 supplement for 4 h; neurons were fixed with 4% paraformaldehyde and stained with 0.1% cresyl violet solution. Images were captured using a VWR microscope, analyzed using NeuronJ, and the number of neurites, equal to or longer than the originating cell body diameter, was calculated with Simple Neurite Tracer plugins for the ImageJ software (NIH).

### Astrocyte/Neuron Co-culture Injury Model

Cortical astrocytes were isolated from C57Bl/6 J newborn mice (P1–3) as previously described [33]. Cells were dissociated in D-MEM/F12 and supplemented with Glutamax, 1% Pen/Strep, and 10% FBS. After 10–12 days in culture, cells were shaken for 24–36 h and treated with 10 mM leucin methylester for 12 h. Astrocyte cultures were then seeded (400,000 cells) onto deformable membrane wells (Bioflex 6 well plates, Flexcell International). The FBS in the medium was reduced after 1 week to 5%, after another 1–2 weeks to 0.5% for 48 h, and then 0% for 6 h. Cells were then mechanically traumatized using an abrupt pressure pulse with a pneumatic device (Flexcell FX-4000 Strain Unit, Flexcell International) programmed to produce a maximal elongation of 23% (130 ms, triangular stretch). After 24 h, cortical neurons were isolated from C57Bl/6 J P5–6 mice as previously described [33]. Treatment with either DV (300 nM) or control (PBS) was performed 6 h after the astrocytes had been stretched. Twenty-four hours post-plating of neurons, cultures were fixed with 4% paraformaldehyde. Neurons and astrocytes were visualized with mouse TuJ1 and chicken GFAP primary antibodies, respectively, and counter-stained with DAPI. To analyze neurite outgrowth, TuJ1-positive cells were digitized using a × 20 objective (Olympus BX51 microscope) and quantified using the ImageJ software (NIH).

### Statistics

All measured variables are presented as mean ± SEM. Analysis of results for comparison between two groups was performed using a Student’s *t* test. For comparisons across multiple groups, a one-way or two-way ANOVA followed by Bonferroni’s or Tukey’s post hoc test was used. Statistical analyses were performed with GraphPad Prism (GraphPad Inc.). A *p* value of < 0.05 is considered significant.

## Results

### DV Levels Are Increased in Human Brain After Stroke

**Fig. 1:**
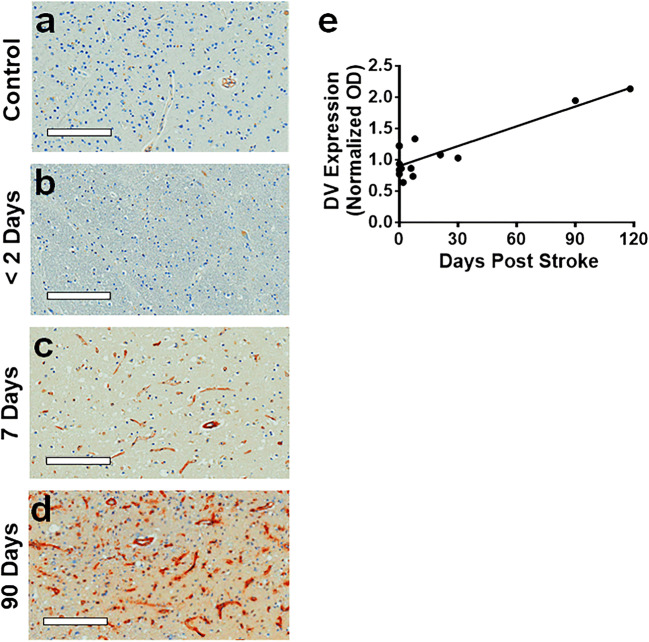
Domain V expression is increased chronically in human stroke brain tissue. **a**–**d** Representative images of DV (brown) immunohistochemistry in human brain tissue counterstained with hematoxylin (blue) from (**a**) control or old infarcted tissue at (**b**) < 2 days, (**c**) 7 days, and (**d**) 90 days. Scale bar = 200 μm. **e** Graph represents quantification (normalized OD) of DV expression (black dots). *n* = 14. Solid line represents linear regression, *R*^2^ = 0.7837, *p* < 0.0001

Clinical relevance of DV to human ischemic stroke was investigated by DV immunohistochemistry on the brains of several stroke patients (Online Resource Table 2) at different post-stroke day (PSD) time points (PSD 1 to PSD 90). DV immunoreactivity was chronically increased in peri-infarct microvessels (Fig. 1).

### Reduced DV Expression Results in Less Neurogenesis After Experimental Stroke

**Fig. 2:**
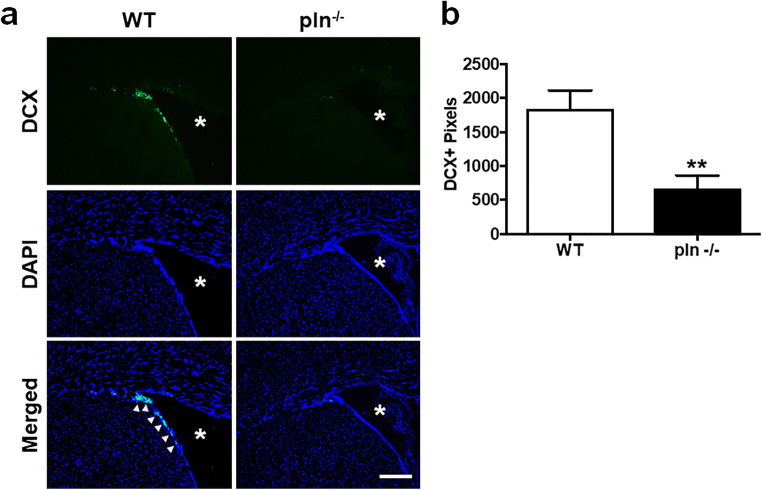
Decreased neurogenesis in pln^−/−^ mice. **a** PSD 14 SVZ representative images of DCX (green) immunofluorescence counterstained with DAPI (blue) in WT and pln^−/−^ mice subjected to MCAo. Arrowheads represent DCX positive staining. White asterisk represents lateral ventricle. Scale bar = 100 μm. **b** Quantification of DCX positive pixels. *P* values were assessed by Student’s *t* test, **p < 0.01 *n* = 5

Pln^−/−^ mice subjected to MCAo showed significantly less doublecortin (DCX) immunoreactivity at the SVZ on PSD 14 compared with WT (*p* < 0.01; Fig. 2a–b), indicating a diminished generation of neuroblast precursor cells at the SVZ that correlates with larger infarcts following MCAo [7]. While there are many steps in the neurogenic process, we will refer to the early neuroblast proliferation and the initial migration out of the SVZ as neurogenesis.

### Delayed Administration of DV Promotes Functional Recovery and Reduces Histological Damage Following Experimental Stroke

DV treatment was delayed until PSD 7 in order to determine whether it could have a broad therapeutic window for experimental stroke as well as to distinguish potential DV effects on neurorepair mechanisms from more acute (e.g., PSD1 to PSD 3) neuroprotective effects [7]. This delayed DV treatment paradigm (Online Resource Fig. 1a) had no adverse effect on mouse weights (Online Resource Fig. 1b) or other observable signs of animal distress.

**Fig. 3:**
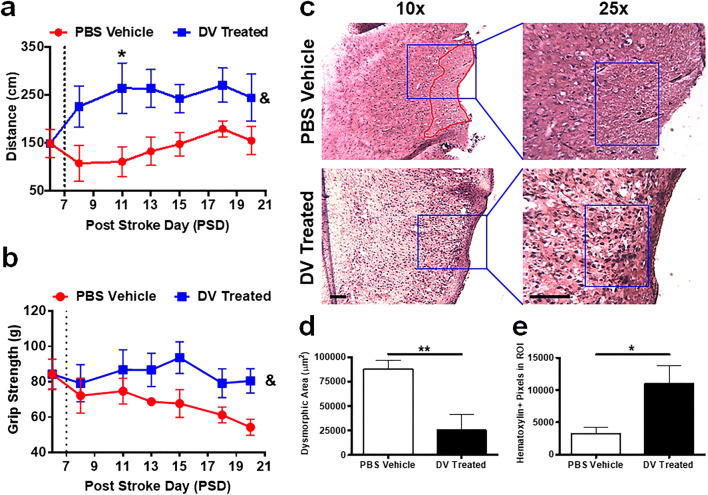
Domain V improves sensorimotor function and nuclear histology in the stroke area. Quantification of (**a**) rotor rod distance (cm) and (**b**) grip strength force (g) behavioral tasks following MCAo with PSD 7 (dashed line) delayed PBS vehicle or DV treatment paradigm. Data for all stroked mice were pooled prior to treatment on PSD 7. **c** PSD 21 representative H&E images at × 10 and for (**d**) dysmorphic area quantification and × 25 for (**e**) hematoxylin-positive pixel quantification. Blue boxes represent regions of interest (ROI) and red outline represents identified dysmorphic areas. Scale bars = 100 μm. *P* values were assessed by two-way RM ANOVA followed by Tukey’s post hoc test (**a** and **b**, *n* = 6) and by Student’s *t* test (**d** and **e**, *n* = 5–7). *p < 0.05, **p < 0.01, & = overall significant effect of treatment

DV-treated mice showed a significant improvement on the rotor rod compared with vehicle-treated, particularly on PSD 11 (*p* < 0.05; Fig. 3a). DV-treated mice also had significantly (*p* < 0.001) better grip strength (Fig. 3b), indicating a marked functional improvement following DV administration.

Next, we assessed histological damage in the ipsilateral cortex to determine if delayed DV treatment influenced brain tissue health. DV treatment reduced the size of hematoxylin and eosin (H&E)-stained dysmorphic areas compared with vehicle-treated stroked controls (*p* < 0.01; Fig. 3c–d) by PSD 21. Of note, 5/7 of the DV-treated animals had no detectable dysmorphic areas, and no indication of contralateral injury was detected in any of the stroked mice (data not shown). Furthermore, DV treatment increased the amount of hematoxylin positive pixels, used as an indicator of nuclei density, within regions of interest compared with vehicle controls (*p* < 0.05; Fig. 3e).

### DV Increases Neurogenesis Following Experimental Stroke

**Fig. 4:**
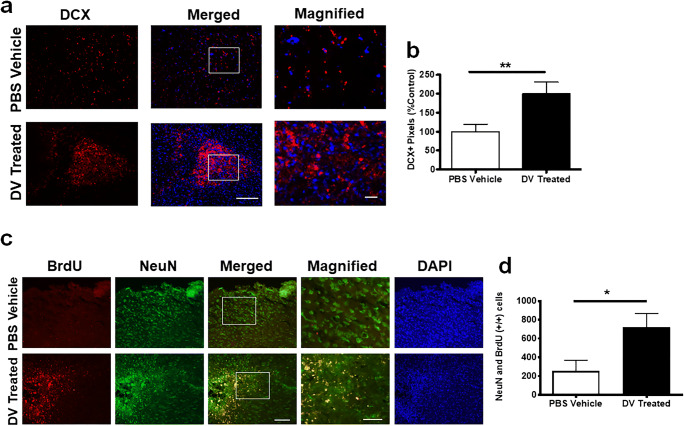
Domain V increases neurogenesis in the infarcted region. **a** Representative PSD 21 images of DCX (red) immunofluorescence counterstained with DAPI (blue) from PBS vehicle and DV-treated MCAo mice. Scale bar = 500 μm. White box indicates region of magnification. Scale bar in magnified image = 100 μm. **b** Quantification of DCX-positive cells, graphed as % of vehicle control, *n* = 15–18. **c** PSD 21 representative images of BrdU (red) and NeuN (green) co-staining (yellow) within the infarct core from PBS vehicle and DV-treated MCAo mice. Scale bar = 100 μm. White box indicates region of magnification. Scale bar in magnified image = 50 μm. **d** Quantification of co-staining (BrdU/NeuN), *n* = 6. *P* values were assessed by Student’s *t* test *p < 0.05 **p < 0.01

As 7-day delayed DV treatment increases cellularity in stroke-affected (i.e., core and peri-infarct) regions on PSD 21, we investigated whether this resulted from increased neurogenesis. DCX immunofluorescence was significantly increased with DV treatment in the stroke-affected region on PSD 21 compared with vehicle-treated mice (*p* < 0.01; Fig. 4a–b). In the uninjured sham controls, DV treatment did not alter DCX immunofluorescence in the SVZ and cortex (Online Resource Fig.2).

Next, we determined whether increased DCX-positive cells associated with DV treatment translated into an increase in new mature neurons in the damaged area following MCAo. We performed co-immunofluorescence with NeuN (pan-neuronal marker) and BrdU (cell proliferation marker) to label neurogenesis after PSD 7. DV-treated mice had significantly higher NeuN- and BrdU-positive co-immunoreactivity in the damaged area (*p* < 0.05; Fig. 4c–d) on PSD 21 compared with vehicle controls.

We next used a second, mechanistically distinct, experimental stroke model of aged mice since neurogenesis diminishes with age [34]. We performed permanent photothrombotic stroke in 24-month-old mice followed by DV treatment at 6 h, previously shown to be neuroprotective and promote functional recovery to PSD 7 [6] when DCX is maximally expressed in this model (data not shown). DV-treated aged mice had significantly higher DCX immunoreactivity in the ipsilateral SVZ on PSD 7 compared with vehicle-treated (Online Resource Fig. 3a–b). At this earlier time point, the majority of DCX-positive cells were identified in, and emerging from, the SVZ rather than near the infarct region itself. Collectively, despite the distinct stroke model, the advanced age of the mice, and the shorter outcome measure (PSD 7 versus PSD 21), DV treatment still appeared to increase mobilization of immature neurons in the stroke-affected brain.

### DV Restores Peri-infarct Excitatory Synaptic Drive to Neocortical Layer 2/3 Pyramidal Cells

**Fig. 5:**
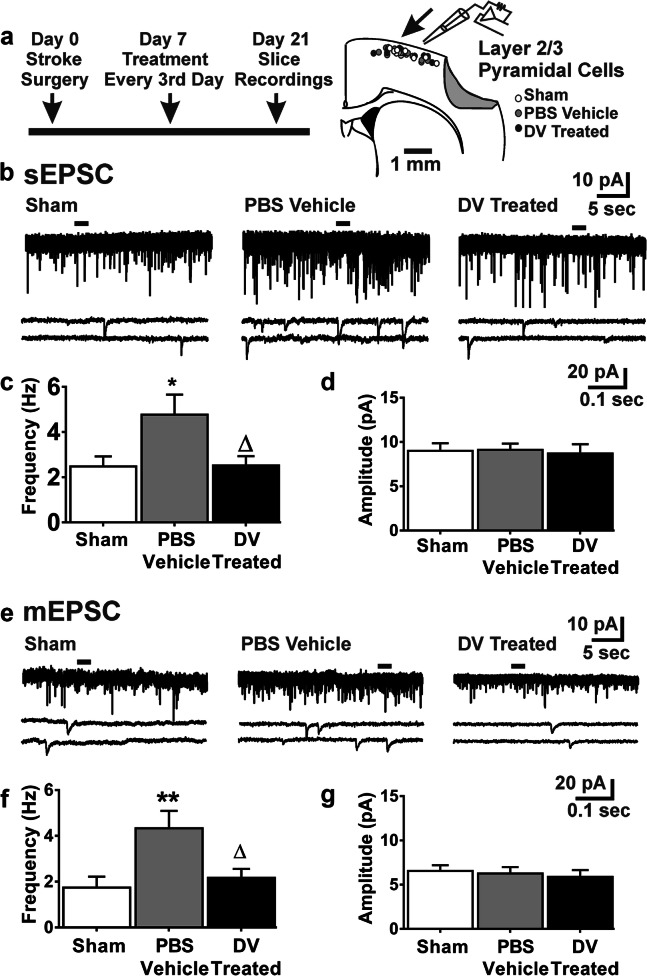
Delayed domain V treatment restores excitatory synaptic drive. **a** Experimental design and schematic of locations of recorded L2/3PCs from mice given sham injury or MCAo with PBS vehicle and DV treatment. **b** PSD 21 representative traces of spontaneous excitatory post-synaptic currents (sEPSCs) for L2/3PCs. Quantification of the sEPSCs (**c**) frequency and (**d**) amplitude. Sham injury *n* = 10, MCAo vehicle-treated *n* = 13, and MCAo DV-treated *n* = 10. **e** PSD 21 representative traces of miniature excitatory post-synaptic currents (mEPSCs) for L2/3PCs. Quantification of the mEPSCs (**f**) frequency and (**g**) amplitude. Sham injury *n* = 8, MCAo vehicle-treated *n* = 9, and MCAo DV-treated *n* = 7. *P* values were assessed by one-way ANOVA followed by Tukey’s post hoc test.*p < 0.05 and **p < 0.01 relative to sham injury. ∆*p* < 0.05 relative to PBS vehicle

We next tested whether delayed DV treatment affected neocortical excitability after stroke. We selected neocortical layer 2/3 pyramidal cells (L2/3PCs) for whole-cell patch-clamp recordings and analysis because of their importance as a predominant site of synaptic integration within the neocortex (Fig. 5a) [35]. As a control measure, no group differences were detected for estimated locations of recorded L2/3PCs based on their relative distance to the most dorsal aspects of both the slice and macroscopic lesion in injured animals (Online Resource Table 3). Membrane potentials were significantly more depolarized in L2/3PCs from stroke injured mice whereas other intrinsic membrane properties of L2/3PCs were not significantly affected by treatment or injury (Online Resource Table 3).

We also examined excitatory synaptic drive to neocortical L2/3PCs, and the frequency of spontaneous excitatory post-synaptic currents (sEPSCs) was significantly elevated in the vehicle-treated stroked animals relative to shams (*p* < 0.05; Fig. 5b–c). This effect was reversed by DV treatment (*p* < 0.05) compared with stroke controls and did not differ from shams. No significant changes were detected in sEPSC amplitude, rise time, or decay time between treatment groups (Fig. 5b, d; Online Resource Table 3).

To better understand these changes in excitatory synaptic drive, we measured signals in the presence of the voltage-gated sodium channel blocker tetrodotoxin (1 μm) in order to examine action potential-independent miniature excitatory post-synaptic currents (mEPSCs), which reflect signaling properties of axon terminals that directly innervate the recorded neuron. As with sEPSCs, the frequency of mEPSCs was significantly increased in cells from vehicle-treated stroked animals (*p* < 0.01) relative to shams (*p* < 0.01; Fig. 5e–f). DV treatment normalized mEPSC frequency in injured animals (*p* < 0.05) when compared with vehicle, with mEPSC frequency not different from shams. No significant changes were detected in mEPSC amplitude, rise time, or decay time between experimental groups (Fig. 5e, g; Online Resource Table 3). Together, these data are consistent with DV exhibiting a restorative effect on synaptic plasticity following ischemic injury.

### DV Increases Neuronal Differentiation and Fetal Cortical Neuron Neurite Extension Through an α2β1 Integrin Dependent Mechanism

**Fig. 6:**
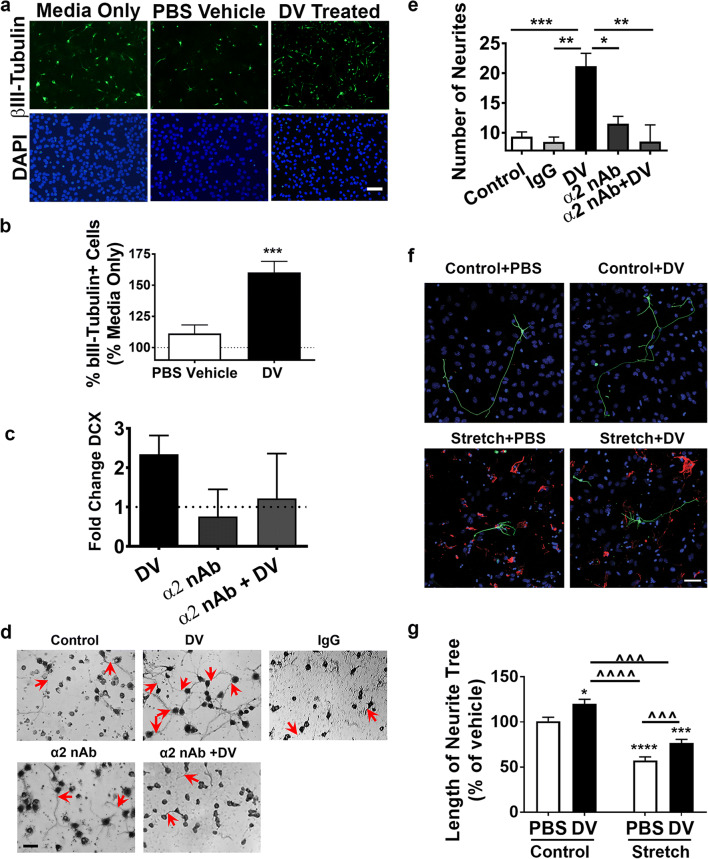
Domain V increases neuronal differentiation of NPC and neurite extension/outgrowth of primary cortical neurons via α2β1 dependent mechanism. **a** NPC representative images and (b) quantification of βIII-tubulin (green) immunofluorescence counterstained with DAPI (blue) from media only, PBS vehicle, and DV-treated conditions. Graphed as % change from media only (dashed line). Scale bar = 100 μm. Results were from 4 independent experiments, with conditions performed in duplicate or triplicate, with 3 images taken per well. *n* = 16. **c** Quantification of DCX fold change compared with vehicle (dashed line). **d** Representative images of cresyl violet stained primary cortical neurons. **e** Quantification of neurite number with either a control IgG or α2 nAb in the presence of PBS vehicle or DV treatment. Scale bar = 10 μm. Results were from 5 total experiments (independent neuronal preparations), with conditions performed in triplicate per experiment, and at least 5 images analyzed per condition. Red arrows represent neurites. **f** Representative images of cortical neurons (TuJ1, green) seeded onto either mature control or stretch-reactive astrocytes (GFAP, red; DAPI, blue). Arrows show the position of the soma while arrowheads represent neurites. Scale bar = 100 μm. **g** Quantification of neurite length, graphed as % PBS control (dashed line). The analysis was performed in three independent co-cultures each performed in triplicate (60–65 neurons total per condition were analyzed). *P* values were assessed using one-way ANOVA followed by Tukey’s post hoc test. *p < 0.05, **p < 0.01, ***p < 0.001, ^^^*p* < 0.001, and ^^^^*p* < 0.0001

We next determined whether DV-induced neuronal differentiation was dependent on α2β1 integrin signaling. On DAP6, DV significantly increased the percent of neurosphere-dissociated cells that differentiated into βIII-Tubulin positive neurons compared with vehicle control (*p* < 0.001; Fig. 6a–b). In addition, qPCR analysis showed that DV increased DCX expression 2.3-fold (Fig. 6c). Under α2 neutralizing antibody (nAb) conditions (26), DCX gene expression decreased compared with vehicle-treated cells, which could not be overcome by DV.

We next assessed DV’s effects on neurite extension (an important step of neurogenesis) using primary mouse fetal cortical neurons (FCN), rather than the mixed population of NPCs. DV significantly enhanced the numbers of neurites after 4 h compared with the media only condition (*p* < 0.001; Fig. 6d–e), which was also blocked by the α2 nAb.

To further test the functional effects of DV on neurite outgrowth, we used an in vitro model of reactive astrogliosis, mechanically stretched cultured astrocytes, which occurs following stroke [36]. DV treatment significantly increased neurite length in both non-stretched (*p* < 0.05) and stretched (*p* < 0.001; Fig. 6f–g) conditions.

### α2β1 Integrin Plays an Important Role in Post-stroke Neurogenesis

**Fig. 7:**
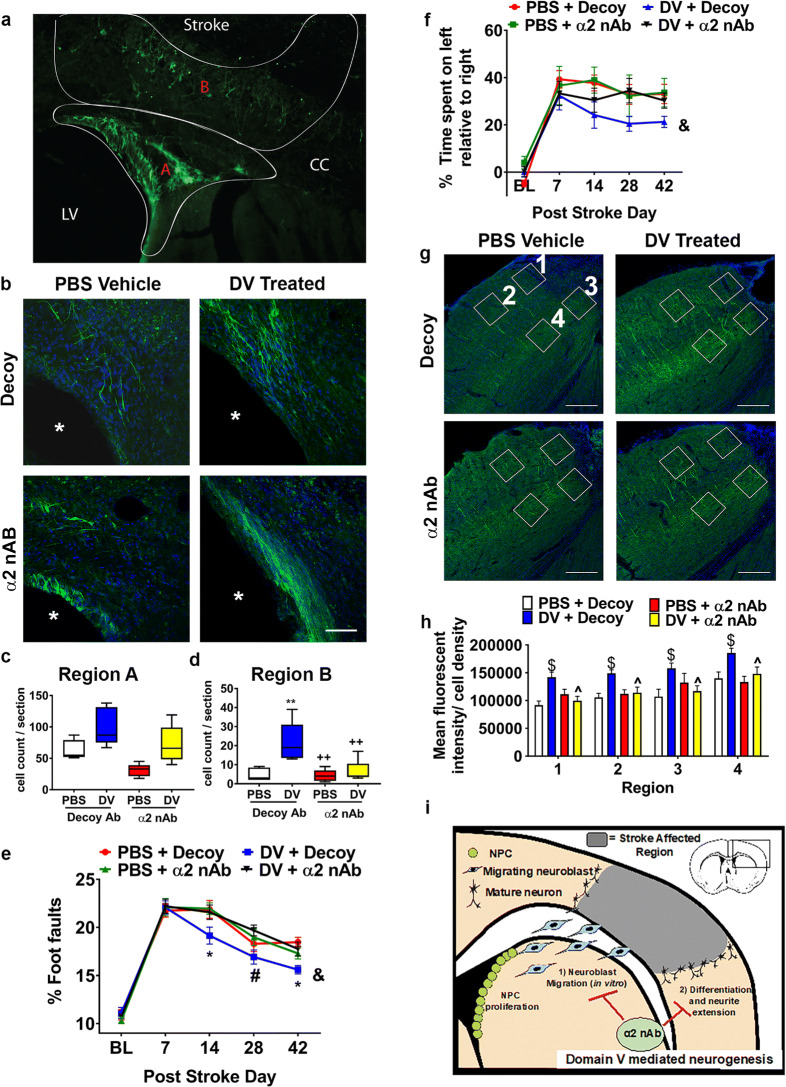
DV increases neurogenesis in photothrombotic stroke model via an α2β1 integrin-dependent mechanism. **a** Sample photomicrograph region A (next to the LV including the bottom half of the corpus callosum (CC)) and region B (the top half of the CC and surrounding the stroke cavity stained with DCX (green)). **b** PSD 42 representative images taken from the dorsal lateral aspect of the LV (white asterisk*) (same as region A) showing DCX (green) immunofluorescence counterstained with DAPI (blue) in α2 or decoy antibody-treated photothrombotic stroked mice that received either PBS vehicle or DV treatment. Quantification of DCX-positive cells in (**c**) region A and (**d**) region B as shown in A, *n* = 5. **e** Quantification of grid walking and (**f**) cylinder behavioral tests, *n* = 7–8. **g** Representative images depicting MAP2 (green) and DAPI (blue) immunofluorescence in the brains of α2 or decoy antibody-treated animals with or without DV treatment. Boxes (150 μm square) show the regions of interest within the peri-infarct that were subjected to analysis from either layer 2/3 (regions 1 & 2) or layer 5 (regions 3 & 4). Quantification of MAP2 staining (**h**) within peri-infarct from regions 1–4 *n* = 8 (data are expressed over cell density). **i** Graphic summary showing DV’s effects on post-stroke neurogenesis. All scale bars = 200 μm. *P* values were assessed using Student’s *t* test or one-way ANOVA followed by Tukey’s post hoc test. *p < 0.05, **p < 0.01 and $*p* < 0.001 compared with PBS + IgG controls and + *p* < 0.05 and ^*p* < 0.001 compared with DV + decoy antibody controls. RM two-way ANOVA was also used (DV + decoy &*p* < 0.05 compared with the other three conditions), *p < 0.05 compared with vehicle + decoy antibody controls (days 14 and 42) and #*p* < 0.05 compared with DV + α2 nAb (day 28)

To investigate the potential role of α2β1 integrin in DV’s post-stroke neurogenic effects in vivo, we administered DV or vehicle 6 h after photothrombotic stroke in young WT mice and then blocked α2β1 integrin with intrathecal treatment of the α2 nAb or IgG control (decoy) on PSD 3. DCX was quantified in two regions of interest; region A is the SVZ of the lateral ventricle and the bottom half of the corpus callosum (CC), and region B is the top half of the CC and surrounding stroke cavity (Fig. 7a–b). While region A had an overall higher abundance of DCX-positive cells, differences between treatments were not significant. However, in region B, mice treated with DV showed a significant increase in DCX-positive cells on PSD 42 compared with control animals (*p* < 0.01; Fig. 7c–d). α2 nAb inhibited DVs neurogenic effect in region B by decreasing the number of DCX-positive cells (*p* < 0.01; Fig. 7d).

We then examined post-stroke functional outcome. Mice treated with DV had a significant improvement in motor skills, measured by grid walk, compared with vehicle at 2 and 6 week post-stroke (*p* < 0.05; Fig. 7e). A significant difference was also observed with the α2 nAb among the DV-treated mice at 42 days post-stroke. Importantly, no difference was observed between mice administered DV + α2 nAb and those given vehicle, suggesting that DV works predominantly through an α2 integrin-mediated mechanism. These effects were also observed in the cylinder test, with DV treatment significantly different than vehicle across time (Fig. 7f). Finally, α2 nAb by itself did not significantly affect functional outcomes after stroke compared with vehicle (Fig. 7e–f).

Next, in parallel to DV’s restorative effects on synaptic plasticity after transient MCAo (Fig. 5), we investigated whether DV might also affect dendrite density in peri-infarct regions after photothrombotic stroke. We found that DV treatment increased (*p* < 0.001) microtubule-associated protein (MAP)2 immunofluorescence in four distinct peri-infarct regions, which was inhibited by α2 nAb (*p* < 0.001, Fig. 7g–h). α2 nAb by itself had no effect on MAP2 immunofluorescence compared with decoy-treated stroked controls (Fig. 7g–h).

## Discussion

In this study, we investigated the role, treatment, and mechanism of action of perlecan DV in post-stroke neurogenesis. We have strengthened the translational and clinical relevance of DV therapy. Perlecan plays an important role in developmental neurogenesis. It is expressed in brain BM [9], the neuroepithelial basal lamina during fetal mouse and human development [10–12], and links angiogenesis and neurogenesis processes [19]. However, to our knowledge, we are the first to demonstrate that perlecan could also play an important role in post-stroke neurogenesis.

In agreement with the particular importance of DV in post-stroke neurogenesis, DV is rapidly and persistently generated in stroked rodent brains, suggesting that it is available at the proper times to impact post-stroke neurogenesis [6, 7], a process that is linked to angiogenesis. Likewise, we demonstrated that DV levels are chronically elevated in human stroke brain tissue (PSD 90). However, it is important to note that the potential influence of age, gender, or the cause of death on post-stroke brain DV levels was not evaluated in our study. Collectively, our studies support the hypothesis that DV is a likely effector of developmental and reparative brain neurogenesis in ischemic stroke.

### Effects of DV on Neocortical Excitability

Whole-cell patch-clamp recordings were used to examine the effect of delayed DV treatment on neocortical excitability. Excitatory synaptic drive to L2/3PCs was increased 21 days after transient MCAo. The increased excitatory synaptic drive within the neocortex is also seen in other brain injury cortical models [37]. Delayed DV treatment reversed these effects and restored excitatory signaling to sham control levels. DV’s restoration of excitatory drive persisted in the presence of TTX and was not paralleled by changes in EPSC amplitude or kinetics, thereby suggesting a local and pre-synaptic mechanism of action. These data provide evidence that DV treatment has neurogenic effects within peri-infarct tissue.

### DV Neurogenesis Effects In Vitro

To investigate the role of DV in neurogenesis, we employed the in vitro neurosphere system. The neurosphere system is a relevant model due to its ability to undergo differentiation into βIII-Tubulin positive neurons [38–40]. One limitation of these and our in vitro neurite extension studies is that we employed cells of fetal or neonatal origin, due to cell robustness and viability, rather than cells derived from the adult mouse brain that might better model our in vivo stroke studies. Therefore, it will be important to confirm our results in adult-derived cells in future studies.

DV increased NPC differentiation into neurons. This finding supports our in vivo results that DV signals new neuroblasts to become new neurons (as indicated by increased BrdU and NeuN co-immunoreactivity in the stroke-affected cortex).

### DV Neuritogenesis Effects In Vitro

Neurite extension is an important process during brain development and neurorepair. Therefore, our finding that DV can support neuronal growth following stretch-induced astrogliosis in vitro is particularly significant as it suggests that delayed DV treatment could promote regeneration. Our results demonstrate that the soluble DV portion of perlecan alone is sufficient to enhance neuritogenesis in vitro and is consistent with our in vivo results demonstrating that post-stroke DV administration increased peri-infarct neurite density.

### DV, α2β1 Integrin, and Neurogenesis

Our results suggest that DV may enhance post-stroke neurogenesis and improve post-stroke functional outcome via the DV α2β1 integrin receptor [7, 8, 41, 42] given that: (1) α2β1 blockade prevents DV-induced post-stroke neurogenic and functional therapeutic benefits in vivo, and (2) blockade of α2β1 integrin prevents DV’s effects on NPC differentiation and FCN neurite extension in vitro (summarized in Fig. 7i). Furthermore, we determined that α2β1 is involved in the NPC-to-neuron differentiation in that the blockade of α2 decreased gene expression of DCX, and DV was not able to reverse this effect. Post-stroke blockade of α2β1 integrin had a greater inhibitory effect on DV-driven neurogenesis on DCX-positive cells in the peri-infarct region versus the peri-ventricular region. This may be due to the experimental design in which α2 nAb was given on PSD 3, while DV treatment was initiated 6 h post-stroke. This could allow for DV to initiate a neurogenic effect with subsequent migration of these cells into the peri-infarct region being blocked by the α2 nAb. Interestingly, α2β1 integrin has been linked to neurite extension in retinal ganglion cells [43], and β1 integrin has been implicated in neurogenesis [43–47].

To our knowledge, we are the first to report the role of α2β1 integrin in post-stroke neurogenesis. Interestingly, both stroked decoy control and α2 nAb-treated mice had very low peri-infarct DCX-positive cell numbers. This, coupled with our observation that α2β1 integrin blockade alone did not worsen functional stroke outcome, could suggest that baseline neurogenesis after photothrombotic stroke may have minimal impact on functional recovery and is susceptible to therapeutic targeting. Alternatively, α2β1 integrin blockade alone could have unknown effects that cumulatively result in unchanged functional stroke outcome.

## Conclusion

Perlecan DV increases neurogenesis and normalizes neocortical excitability in vivo after experimental stroke in young and aged mice as well as in some stages of neurogenesis in vitro. This effect coincides with improved functional outcomes, even after delayed initiation of treatment, suggesting a potentially broad therapeutic window for DV. These effects may be mediated, in part, by α2β1 integrin, a receptor that we have also demonstrated to play a key and previously unrecognized role in post-stroke neurogenesis.

While perlecan has been implicated in developmental neurogenesis, our study demonstrates that it is also important for post-stroke neurogenesis and that its small DV portion could be used as an exogenous stroke therapy with a clinically relevant broad therapeutic window.

## Electronic Supplementary Material

ESM 1 (DOCX 1337 kb)

## Data Availability

The data that support the findings of this study are available from the corresponding author upon reasonable request
