# Supplementary material for: Perlecan Domain-V Enhances Neurogenic Brain Repair After Stroke in Mice
Source: Transl Stroke Res. 2020 Apr 7;12(1):72–86. doi: 10.1007/s12975-020-00800-5 (PMC7803718; doi:10.1007/s12975-020-00800-5)
Supplement: Supplementary file 1 — (DOCX 1337 kb) [file 12975_2020_800_MOESM1_ESM.docx]

**Perlecan Domain-V Enhances Neurogenic Brain Repair after Stroke in Mice**

Amanda L. Trout^1,5*^, Michael P. Kahle^1,11*^, Jill M. Roberts^1,2^, Aileen Marcelo^1^, Leon de Hoog^1^, Jeffery A. Boychuk^3^, Stephen L. Grupke^4^, Antonio Berretta^6^, Emma K. Gowing^6^, Carie R. Boychuk^3^, Amanda A. Gorman^1^, Danielle N. Edwards^1,2^, Ibolya Rutkai^7,8^, Ifechukwude J Biose^7^, Hatsue Ishibashi-Ueda^9^, Masafumi Ihara^10^, Bret N. Smith^2,3^, Andrew N. Clarkson^6^, and Gregory J. Bix^1,2,4,5,7,8^

^1^Sanders-Brown Center on Aging, University of Kentucky, Lexington, Kentucky, USA.

^2^Department of Neuroscience, University of Kentucky, Lexington, Kentucky, USA.

^3^Department of Physiology, University of Kentucky, Lexington, Kentucky, USA.

^4^Department of Neurosurgery, University of Kentucky, Lexington, Kentucky, USA.

^5^Department of Neurology, University of Kentucky, Lexington, Kentucky, USA.

^6^Department of Anatomy, Brain Health Research Center and Brain Research New Zealand,

University of Otago, Dunedin, New Zealand.

^7^Clinical Neuroscience Research Center, Department of Neurosurgery, Tulane University School of Medicine, New Orleans, LA, USA

^8^Tulane Brain Institute, Tulane University, New Orleans, LA, USA

^9^Department of Pathology, National Cerebral and Cardiovascular Center, Suita, Japan

^10^Department of Neurology, National Cerebral and Cardiovascular Center, Suita, Japan.

^11^Department of Neuroscience and Experimental Therapeutics, Texas A&M Health Science

Center College of Medicine, Bryan, Texas, USA.

* These authors contributed equally to this work.

Corresponding author current address:

Gregory J. Bix, M.D., Ph.D., F.A.H.A.

Tulane University School of Medicine

Center for Clinical Neurosciences

131 S. Robertson

Ste 1300, Room 1349

New Orleans, LA 70112

ORCID iD: 0000-0002-8969-9553

e-mail: gbix@tulane.edu

Tel: 504-988-3564


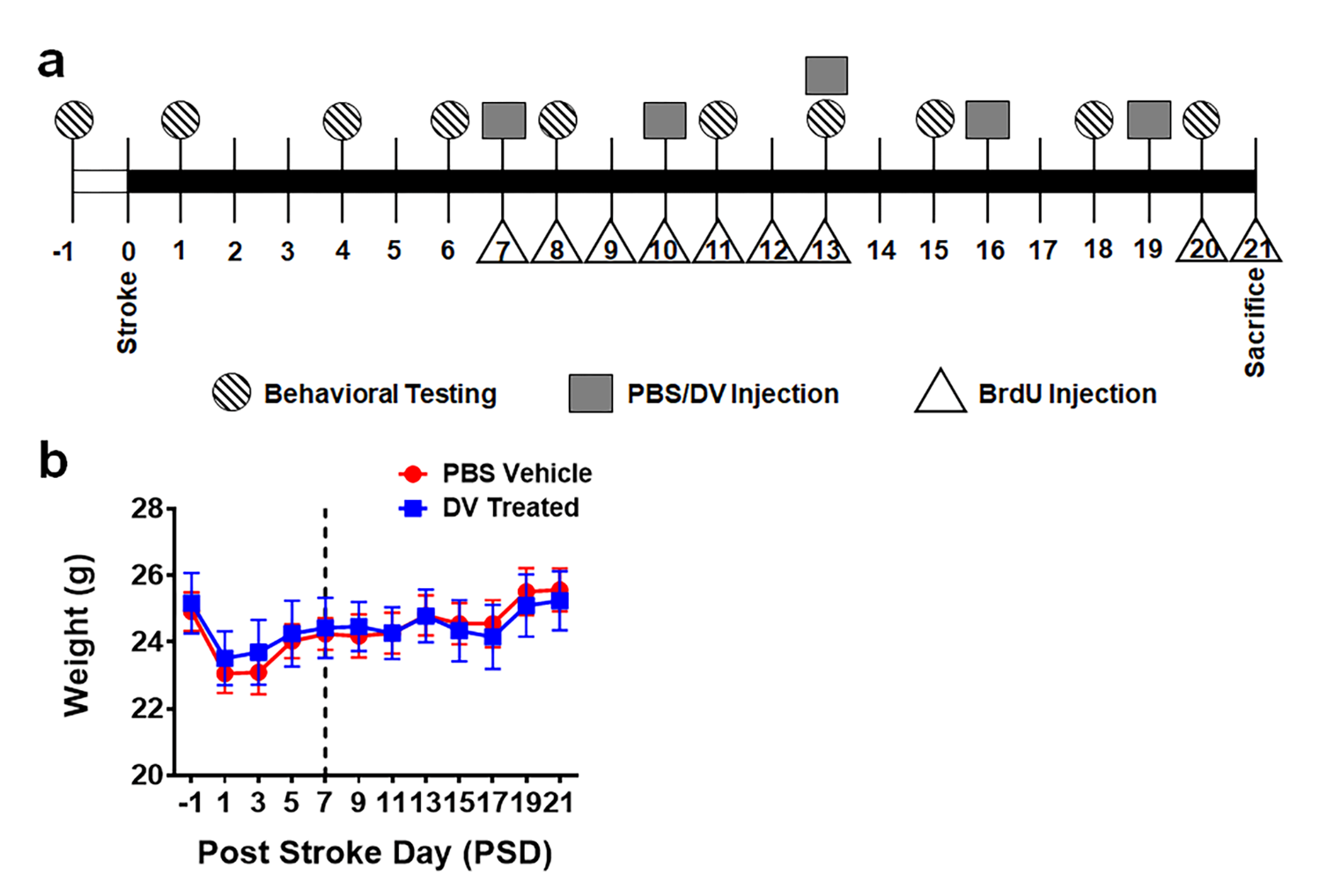


**Online Resource 1** (a) Experimental timeline and (b) animal weights (g; n=7) for MCAo with PSD 7 delayed DV treatment paradigm.


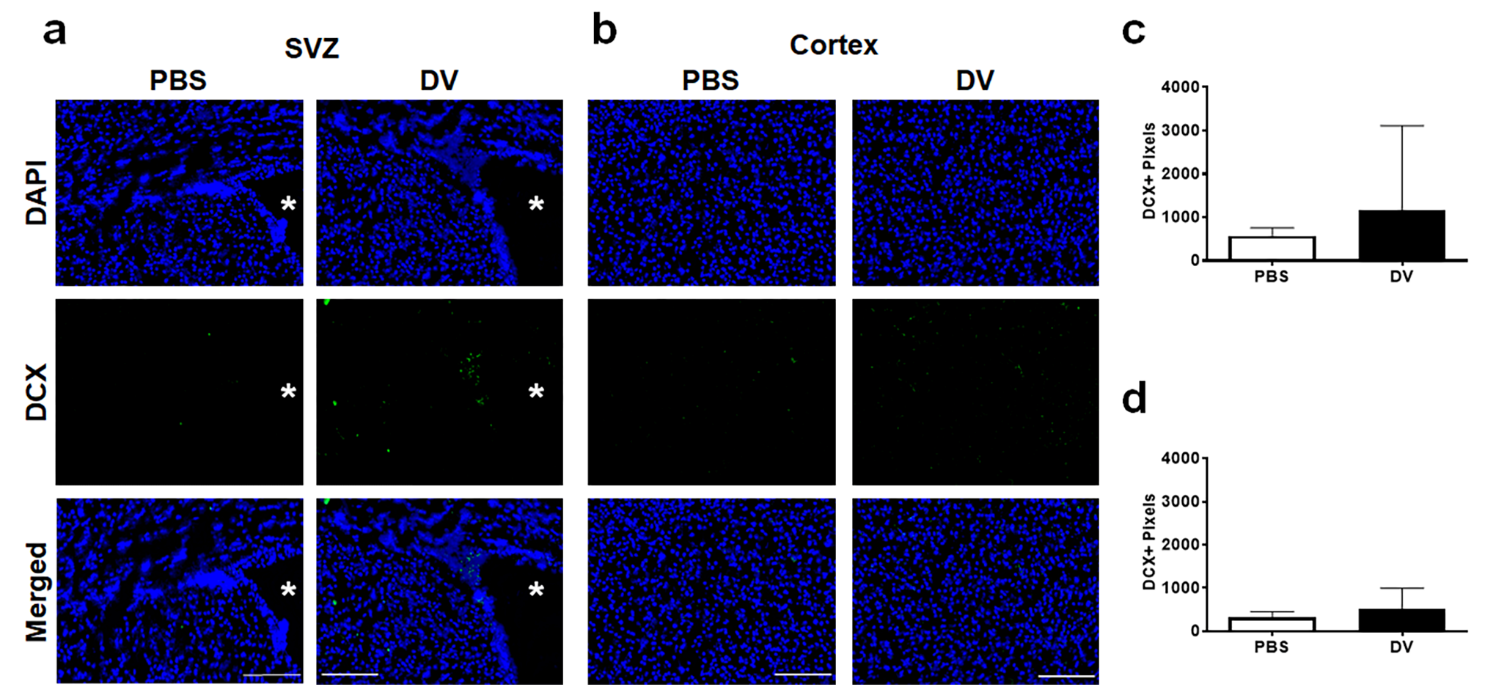


**Online Resource 2** Sham treated PBS and DV representative PSD 14 images of DCX (green) immunofluorescence counterstained with DAPI (blue) within the (a) SVZ and (b) cortex. White * represents lateral ventricle. Scale bar = 100μm. Quantification (n=4) of DCX positive pixels in the (c) SVZ and (d) cortex. Data presented as mean ± SD. P values were assessed using Students t-test.


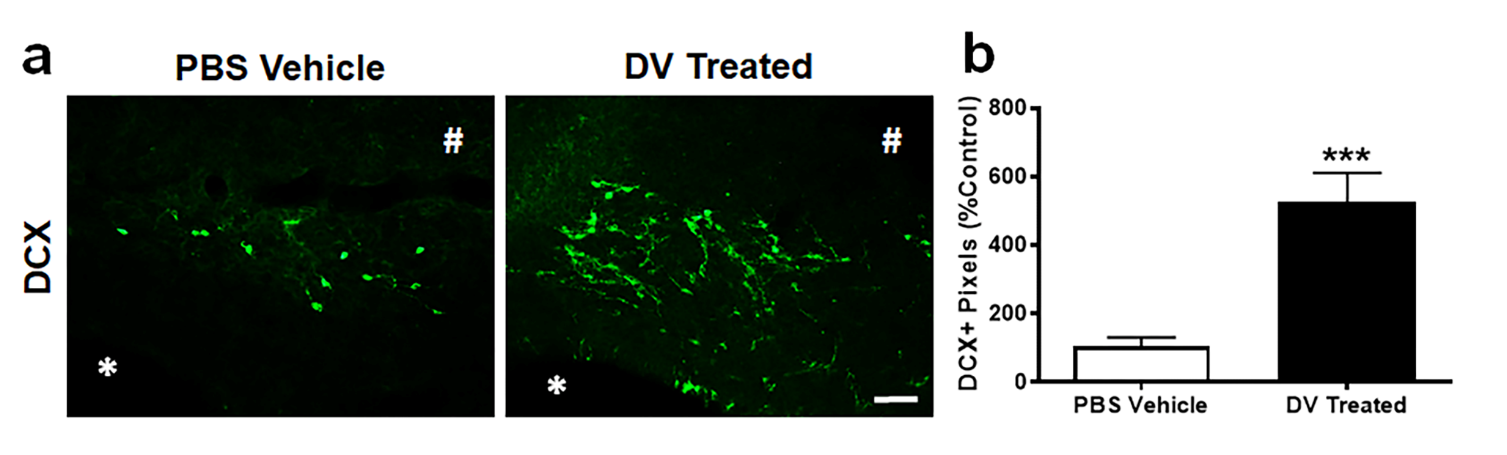


**Online Resource 3** Domain V increases number of DCX positive neurons in aged mice. (a) PSD 7 representative images of DCX (green) immunofluorescence from aged mice subject to photothrombotic stroke. White * indicates SVZ and # indicates infarct region. Scale bar = 50μm (b) DCX-positive pixel quantification (% of vehicle control). P values were assessed by Student’s t-test. ***p<0.001. n=6-7.

**Online Resource Table 1** Primary antibody list.

| Antibody | Company | Cat. No |
| --- | --- | --- |
| Mouse anti-human endorepellin/Perlecan DV | R&D Systems | MAB2364 |
| Rabbit anti-DCX | Abcam | ab18723 |
| Mouse anti-NeuN | Abcam | ab104224 |
| Rabbit anti-βIII-Tubulin | Abcam | ab18207 |
| Rabbit anti-MAP2 | Millipore | Ab5622 |
| Rat anti-BrdU | Abcam | ab6326 |
| Mouse anti-TuJ1 | R&D Systems | NL1195R |
| Chicken anti-GFAP | Millipore | AB5541 |

**Online Resource Table 2** Clinical data of human ischemic stroke patients.

| **Patient ID** | **Sex** | **Age** | **Stroke Onset to Death (Days)** | **Clinical Diagnosis** |
| --- | --- | --- | --- | --- |
| Control 1 | M | 78 | N/A | congestive heart failure |
| Control 2 | M | 41 | N/A | idiopathic pulmonary hypertension |
| Control 3 | M | 60 | N/A | Guillain-Barré syndrome, hemorrhagic shock by intraperitoneal hemorrhage |
| Control 4 | M | 38 | N/A | left isomerims, double-outlet right ventricle |
| Control 5 | M | 70 | N/A | hypokalemic myopathy, QT prolongation |
| 1 | M | 71 | 1 | basilar artery occlusion |
| 2 | F | 88 | 2 | systemic emboli/ paroxysmal atrial fibrillation |
| 3 | M | 62 | 6 | old myocardial infarction / coronary artery bypass graft / bladder cancer / suspect adrenal tumor |
| 4 | F | 77 | 7 | heart failure / pneumonia |
| 5 | F | 89 | 7-8 | right internal carotid artery occlusion / suspect cardio embolic stroke |
| 6 | M | 78 | 21 | deep venous thrombosis / bilateral internal carotid artery occlusion / abdominal aortic aneurysm |
| 7 | F | 93 | 30 | MRSA-pneumonia |
| 8 | M | 56 | 90 | myocardial infarction / disseminated intravascular coagulation / suspect lung cancer / gastrointestinal bleeding |
| 9 | F | 75 | 118 | cerebral infarction, cerebral amyloid angiopathy |

**Online Resource Table 3** Location and intrinsic and excitatory synaptic properties of neocortical layer 2/3 pyramidal cells (L2/3PCs) *ex vivo*.

|  | **Experimental Group** | | | |
| --- | --- | --- | --- | --- |
|  | **Sham-injury** | **Injury + PBS** | **Injury + DV** | **Statistical Test** |
| **Dorsal-medial aspect of slice (µm)** | 2241.5 ± 174.8 | 2116.6 ± 183.9 | 2137.0 ± 264.7 | F(2,31)= 0.11, p=0.90 |
| **Dorsal-lateral aspect of lesion (µm)** | _____ | 1098.2 ± 138.6 | 1049.4 ± 230.6 | T(21)= 0.19, p=0.85 |
| **Resting Membrane Potential** | -67.2 ± 2.5 | -58.4 ± 2.0  vs sham* (**p=0.021**) | -56.8 ± 2.2  vs sham* (**p=0.0098**) | F(2,31)= 6.00  * **p=0.0062** |
| **Input Resistance (MΩ)** | 256.8 ± 48.1 | 246.9 ± 48.9 | 323.4 ± 54.5 | F(2,31)= 0.65, p=0.53 |
| **AP Threshold (mV)** | -33.7 ± 0.9 | -34.0 ± 1.2 | -32.8 ± 1.4 | F(2,31)= 0.24, p=0.78 |
| **sEPSC Frequency (Hz)** | 2.5 ± 0.4 | 4.8 ± 0.8 | 2.6 ± 0.4 | F(2,30)= 4.40,  * **p=0.021** |
| **sEPSC Amplitude (pA)** | 9.1 ± 0.7 | 9.2 ± 0.6 | 8.8 ± 0.9 | F(2,30)= 0.08, p=0.93 |
| **sEPSC Rise (ms)** | 2.2 ± 0.06 | 2.1 ± 0.18 | 2.4 ± 0.13 | F(2,30)= 0.69, p=0.51 |
| **sEPSC Decay (ms)** | 3.1 ± 0.20 | 3.2 ± 0.32 | 3.7 ± 0.29 | F(2,30)= 1.23, p=0.31 |
| **mEPSC Frequency (Hz)** | 1.8 ± 0.4 | 4.4 ± 0.7 | 2.2 ± 0.4 | F(2,21)= 6.52,  * **p=0.0063** |
| **mEPSC Amplitude (pA)** | 6.7 ± 0.5 | 6.4 ± 0.6 | 6.0 ± 0.6 | F(2,21)= 0.29, p=0.75 |
| **mEPSC Rise (ms)** | 2.4 ± 0.22 | 2.0 ± 0.20 | 2.3 ± 0.10 | F(2,21)= 1.43, p=0.26 |
| **mEPSC Decay (ms)** | 3.4 ± 0.46 | 2.9 ± 0.38 | 3.4 ± 0.37 | F(2,21)= 0.44, p= 0.65 |

* p<0.05
